# Convolutional recurrent U‐net for cardiac cine MRI reconstruction via effective spatio‐temporal feature exploitation

**DOI:** 10.1002/mp.70245

**Published:** 2025-12-31

**Authors:** Donghang Lyu, Marius Staring, Matthias J. P. van Osch, Mariya Doneva, Hildo J. Lamb, Nicola Pezzotti

**Affiliations:** ^1^ Department of Radiology Leiden University Medical Center Leiden the Netherlands; ^2^ Cardiologs Philips Paris France; ^3^ Faculty of Computer Science Eindhoven University of Technology Eindhoven the Netherlands; ^4^ Philips Innovative Technologies Hamburg Germany

**Keywords:** cardiac cine MRI reconstruction, convolutional recurrent U‐Net, spatio‐temporal feature

## Abstract

**Background:**

Cardiac Cine Magnetic Resonance Imaging (MRI) provides dynamic visualization of the heart's structure and function but is hindered by slow acquisition, requiring repeated breath‐holds that challenge sick patients. Accelerated imaging can mitigate these issues but potentially reduce spatial and temporal resolution. Therefore, innovative approaches are essential to ensure effective performance under high acceleration conditions. Deep learning‐based reconstruction methods show promise in enhancing image quality from highly undersampled data, accelerating scans while maintaining diagnostic accuracy. However, they often fail to effectively exploit the spatio‐temporal features inherent to cine MRI, which are essential for accurate reconstruction, thereby leaving room for further improvement.

**Purpose:**

We aim to more effectively exploit the spatio‐temporal features inherent in cine MRI sequences by integrating convolutional recurrent operations with a U‐Net architecture, enhancing the reconstruction performance of cine MRI.

**Methods:**

We developed a new deep learning model called **CRUNet‐MR** that enhances the extraction of spatio‐temporal features by combining convolutional recurrent operations with a U‐Net structure. This design ensures continuous extraction of temporal features while fusing fine‐grained spatial details with high‐level semantic information. Furthermore, dilated convolutions are incorporated to expand the spatial receptive field, and appropriate combinations of dilation factors are explored to further enhance overall performance.

**Results:**

Training, validation, and testing were performed on the public CMRxRecon2023 dataset, using two views and four acceleration factors ranging from 4 to 24 with the given Auto‐Calibration Signal (ACS) area. The dataset consists of 120 subjects for training, 60 for validation, and 120 for testing. In general, the proposed CRUNet‐MR shows statistically significant differences with benchmark models and consistently outperforms them, particularly showcasing better reconstruction quality in dynamic regions, highlighting its effective extraction of spatio‐temporal features. Ablation studies further validated the design choices of CRUNet‐MR. The model demonstrated strong reconstruction performance, achieving an average SSIM of 0.986 at an acceleration factor of 4 and 0.971 at a factor of 8 across both views. Furthermore, CRUNet‐MR was validated on a small in‐house LUMC dataset, showing its generalization capability and rapid adaptability through fine‐tuning.

**Conclusions:**

The proposed CRUNet‐MR model is well‐suited for cine MRI reconstruction, effectively leveraging spatio‐temporal features to reconstruct high‐quality images, especially in dynamic cardiac regions. This capability highlights its potential to support higher acceleration factors, enabling faster and more patient‐friendly cardiac imaging.

## 1 INTRODUCTION

Cardiac magnetic resonance (CMR) imaging stands as a robust, non‐invasive modality for comprehensive evaluation of cardiac structure, function, and blood flow. Within this realm, Cine MRI plays a pivotal role by capturing the dynamic motion of the heart, providing crucial insights into cardiac physiology and aiding in the diagnosis of various cardiovascular conditions.^1^ However, cine MRI is still hindered by the lengthy process of acquiring *k*‐space data over multiple cardiac cycles. This extended acquisition time necessitates patients remaining still inside the scanner bore, as even slight movements can lead to slice misalignment,^2^ which reduces image quality and diagnostic accuracy. Patients must also repeatedly hold their breath during scanning to minimize motion artifacts, which can be both challenging and uncomfortable.^3^ The use of high acceleration factors offers a reduction in the number of breath‐holds, saving time for both patients and clinicians.^4^ However, this would reduce temporal and spatial resolution, which limits its practical applicability.^5^

Various techniques have been developed to reduce scan time in MRI, including parallel imaging, which reconstructs missing data using information from multiple coils, and compressed sensing, which utilizes sparsity transforms and iterative algorithms for image recovery. In all these cases, acceleration is achieved by acquiring a fraction of the *k*‐space data, and applying advanced reconstruction techniques to restore the image quality. Cardiac cine MRI includes an additional temporal dimension capturing the heart at different motion states. Therefore, there is a strong spatio‐temporal correlation across the cine sequence due to the high predictability and similarity between consecutive cardiac frames. These temporal dynamics and inter‐frame dependencies can be exploited by reconstruction methods to enhance the overall performance of cine MRI, requiring more sophisticated approaches than those used in conventional MRI. Some traditional works^6^, ^7^, ^8^ have explored the spatio‐temporal correlations for reconstructing undersampled cardiac cine MRI data. They combine the concepts of parallel imaging and compressed sensing to exploit the spatio‐temporal correlation in cine MRI, applying temporal averaging operation based on the overall similarity of the whole cine sequence. Additionally, some low‐rank methods^9^, ^10^ are proposed to minimize the sparsity of high‐frequency components and the rank of the low‐rank structure of the temporal data. Although some progress has been made in cine MRI reconstruction by these methods, there are still some limitations, particularly when it comes to pushing the boundaries of higher scan acceleration.

In recent years, deep learning has emerged as a promising approach for MRI reconstruction, gaining more attention in cardiac cine MRI reconstruction as well. Deep learning methods aim to further enhance reconstruction performance by effectively handling complex artifacts and enabling higher acceleration factors. Compared to traditional regularization terms in the reconstruction pipeline, which rely on explicit assumptions, deep learning methods can replace and improve upon them through learning from the data, allowing the physics‐based data consistency step to better align with the acquired measurements. Moreover, these models adapt to diverse data, recovering key features while preserving image quality even in the presence of significant noise and artifacts. Initially, early models for cine MRI reconstruction employed basic convolutional neural networks (CNNs) with 3D^11^, ^12^ or (2+1)D convolutional^11^ layers to extract spatio‐temporal features in an unrolled design. However, these convolution operations primarily focus on local temporal and spatial features, which limits the model's ability to capture critical global information across the entire cine sequence. Moreover, some deep learning methods applied these convolutional operations in the frequency domain instead of the image domain. For instance, CineNet^13^ employs a lightweight CNN‐based U‐Net^14^ in the temporal Fourier domain by applying the Fourier transform along the temporal dimension to extract global temporal features, followed by a conjugate gradient (CG) method as a data consistency module. However, the simplicity of the applied U‐Net model and the use of the temporal Fourier domain as input may limit its ability to effectively exploit spatio‐temporal information.

As a pioneering deep learning model for cardiac cine MRI reconstruction, CRNN‐MRI^15^ was the first method to introduce convolutional recurrent operations across frames and iterations. By extracting spatio‐temporal features throughout the sequence and providing valuable information for subsequent iterations, it achieves strong performance and demonstrates its potential in this field. Despite its effectiveness, CRNN‐MRI has some limitations that hinder its performance. The model restricts continuous temporal feature exploration in the cine sequence by applying recurrent operations only once at the start of each cascade block, rather than throughout the entire network. Fully exploiting the spatio‐temporal features of cine MRI is essential, in particular, for accurately reconstructing the dynamic regions of the heart. Furthermore, the shallow depth of the network and the small convolutional kernel size limit the spatial receptive field, potentially impacting its ability to capture high‐level spatial information. Although subsequent works^16^, ^17^ build upon the same convolutional recurrent operations by either extracting additional domain‐specific information through multiple convolutional recurrent branches or recovering *k*‐space data before inputting it into the CRNN‐MRI model, the fundamental limitations of the CRNN‐MRI architecture remain unresolved. Then, a recent model, PromptMR^18^ introduced a novel strategy for leveraging spatio‐temporal correlations in cine MRI sequences. It takes five consecutive frames as input to reconstruct the central frame, which inherently limits the temporal context by excluding more distant frames. To capture spatio‐temporal correlations from a global perspective, PromptMR merges the temporal and channel dimensions and applies channel attention to the resulting representation. Additionally, the use of learnable prompts in the decoder enhances the model's adaptability to varying image contrasts. Despite its strong performance in cardiac cine MRI reconstruction, PromptMR does not exploit potentially informative distant frames, which could provide valuable context for reconstruction. Furthermore, reconstructing only one frame at a time prolongs the overall processing time.

Despite advancements in existing methods, effectively leveraging spatio‐temporal features for improved reconstruction remains a challenge and requires further investigation. Although the attention mechanism from transformer^19^ offers powerful global feature extraction, its high resource demands limit integration into unrolled architectures for full cine sequence processing. In contrast, convolutional recurrent operations are both effective for capturing spatio‐temporal features and well‐suited for use within unrolled designs, which was also supported by the CRNN‐MRI paper.^15^ The convolutional component effectively extracts spatial features from each frame, while the recurrent component facilitates information propagation across frames, naturally diminishing the influence of distant frames. Consequently, convolutional recurrent operations are capable of extracting both local and global spatio‐temporal features effectively. In this work, we present **CRUNet‐MR**, an unrolled network that effectively integrates the convolutional recurrent operations within a U‐Net structure in each cascade block. The U‐Net structure is known for its ability to merge fine‐grained spatial details with high‐level semantic information through its skip connections, making it a widely adopted and proven design for reconstruction tasks. Therefore, CRUNet‐MR aims to maintain continuous extraction of spatio‐temporal features within the sequence while effectively integrating multi‐level features, ultimately enhancing cine MRI reconstruction performance by better exploiting the spatio‐temporal features inherent in the cine sequence. The key contributions of CRUNet‐MR are as follows:

1. CRUNet‐MR effectively combines convolutional recurrent operations with the U‐Net structure. By splitting a bidirectional convolutional recurrent unit into two convolutional recurrent units with opposite directions and integrating them into the U‐Net structure, it enables continuous extraction of spatio‐temporal features within the sequence and efficient information propagation across cascade blocks.
2. Extensive ablation studies examine the introduced model components, loss terms, and different dilation factor combinations, demonstrating the contribution of each designed element.
3. By comparing against benchmark models on the CMRxRecon2023 dataset and analyzing the reconstruction performance of the dynamic region of the heart, CRUNet‐MR demonstrates strong performance across various acceleration factors and views, effectively enhancing spatio‐temporal feature exploitation within the cine sequence.

## 2 METHODS

### 2.1 Problem formulation

In general, cine MRI reconstruction is fundamentally an image‐formation problem governed by the MRI forward model and acquisition physics. Given a complex‐valued cine MRI image series $x\in C^{T\times D_{h}\times D_{w}}$ from multi‐coil undersampled *k*‐space data y, where T denotes the number of time frames, and $D_{h}$ and $D_{w}$ represent the height and width of each frame, respectively, the goal of cine MRI reconstruction can be formulated as follows:

(1) $$\underset{x}{\mathrm{argmin}}{∥y-Ax∥}_{2}^{2}+\lambda R\left(x\right),$$

where A represents the linear forward operator, consisting of the coil sensitivity map S, the 2D Fourier transform F, and the undersampling mask M. The term R denotes the regularization, with λ as a hyper‐parameter controlling its strength. For deep learning methods, R represents a trainable neural network. Following the ADMM^20^ optimization algorithm, an intermediate variable z is introduced. When constraining z to be equal to x, the above problem is reformulated as

(2) $$\underset{x,z}{\mathrm{argmin}}{∥y-Ax∥}_{2}^{2}+\mu {∥x-z∥}_{2}^{2}+\lambda R\left(z\right).$$

In an unrolled network, the above formula can be solved iteratively using the following procedure:

(3) $$z^{i}=\underset{z}{\mathrm{argmin}}\lambda R\left(z\right)+\mu {∥x^{i}-z∥}_{2}^{2},$$

(4) $$x^{i+1}=\underset{x}{\mathrm{argmin}}{∥y-Ax∥}_{2}^{2}+\mu {∥x-z^{i}∥}_{2}^{2}.$$

Here, $x^{i}$ is processed by the ith cascade block, represented by a neural network $N_{\theta }^{i}$, to produce $z^{i}$, which is subsequently used to generate the input for the next cascade block, $x^{i+1}$. Equation (4) can be interpreted as a data consistency (DC) operation within the reconstruction pipeline. This operation preserves the sampled *k*‐space values, ensuring the reconstructed output remains consistent with the original undersampled *k*‐space data. The details of the DC operation are outlined as follows:

(5) $$x^{i+1}=DC\left(z^{i},y,\lambda_{0},\Omega \right)=A^{†}\Lambda Az^{i}+\frac{\lambda_{0}}{1+\lambda_{0}}A^{†}y,$$

(6) $$\Lambda_{kk}=\left\{\begin{array}{ccc} & 1 & if\;k\notin \Omega \\ & \frac{1}{1+\lambda_{0}} & if\;k\in \Omega \end{array}\right.,$$

where $A^{†}$ denotes the Hermitian operation of A, $\lambda_{0}$ is a regularization parameter, Ω represents the index set of the acquired *k*‐space data, and Λ is a diagonal matrix. Its diagonal values are 1 for indices outside the acquired *k*‐space area, and $\frac{1}{1+\lambda_{0}}$ for indices within the acquired area. As $\lambda_{0}$ approaches infinity, this indicates maintaining the original sampled *k*‐space area while interpolating the zero‐filled regions using the neural network output.

In the CRUNet‐MR reconstruction pipeline, the model is formulated as a learned proximal solver for the inverse problem described in Equation (1). Within each cascade block, the regularization operator R is implemented as a learned prior via the CRUNet module. The network alternates between (i) a learned denoising or proximal step in the image domain and (ii) a physics‐based data consistency step that projects the reconstruction back into *k*‐space to enforce agreement with the acquired measurements. Moreover, the model is trained on complex‐valued data, incorporating both k‐space and image‐domain loss terms to jointly preserve quantitative fidelity and perceptual quality.

### 2.2 Convolutional recurrent units

As mentioned earlier, the CRNN‐MRI model employs two kinds of convolutional recurrent operations that learn representations across both the temporal dimension of the cine sequence and cascade blocks: Bidirectional convolutional recurrent units evolving over time and iterations (BCRNN‐TI) and convolutional recurrent units evolving over iterations (CRNN‐I). In this context, “*iteration*” refers to each cascade block, highlighting the iterative nature of the unrolled model. In the CRUNet‐MR model, we solely incorporate the convolutional recurrent operation evolving over time and iterations to ensure the efficient extraction of spatio‐temporal features.

For the BCRNN‐TI block, its overall working principle can be depicted as follows:

(7) $$F_{l}^{i}={\text{BCRNN-TI}}_{\theta }\left(F_{l-1}^{i},F_{l}^{i-1},H_{l,0}^{i}\right).$$

In the lth layer of ith cascade block, the BCRNN‐TI block receives three inputs: (1) the initial hidden state $H_{l,0}^{i}$, initialized as a zero matrix; (2) the output feature map from the previous layer of the same block $F_{l-1}^{i}$; and (3) the output feature map from the same layer of the previous cascade block $F_{l}^{i-1}$. In this context, $F_{l-1}^{i}$ and $F_{l}^{i-1}$ offer some prior information from different perspectives. Meanwhile, the hidden state $H_{l,0}^{i}$ plays a crucial role in connecting the frames of sequence by preserving contextual information for each input element. Then more details about its internal working process are illustrated as follows:

(8) $$H_{l,t}^{i}={\vec{H}}_{l,t}^{i}+{\overset{\leftarrow }{H}}_{l,t}^{i},$$

(9) $${\vec{H}}_{l,t}^{i}=\sigma \left(W_{l}*H_{l-1,t}^{i}+W_{t}*{\vec{H}}_{l,t-1}^{i}+W_{i}*H_{l,t}^{i-1}+{\vec{B}}_{l}\right),$$

(10) $${\overset{\leftarrow }{H}}_{l,t}^{i}=\sigma \left(W_{l}*H_{l-1,t}^{i}+W_{t}*{\overset{\leftarrow }{H}}_{l,t+1}^{i}+W_{i}*H_{l,t}^{i-1}+{\overset{\leftarrow }{B}}_{l}\right),$$

(11) $$F_{l,t}^{i}=\text{concat}\left(H_{l,1}^{i},H_{l,2}^{i},\text{…},H_{l,n-1}^{i},H_{l,n}^{i}\right).$$

For the hidden state at the lth layer, the tth frame, and the ith cascade block, $H_{l,t}^{i}$ is the summation of ${\vec{H}}_{l,t}^{i}$ and ${\overset{\leftarrow }{H}}_{l,t}^{i}$. The right arrow denotes the forward propagation of hidden states, where information flows from the previous frame to the current frame, while the left arrow represents backward propagation. Then, each of them is computed as the sum of the convolutional results of the hidden state from previous layer $H_{l-1,t}^{i}$, the hidden state of the next frame ${\overset{\leftarrow }{H}}_{l,t+1}^{i}$ (or the previous frame ${\vec{H}}_{l,t-1}^{i}$), and the hidden state from the previous cascade block $H_{l,t}^{i-1}$, followed by applying an activation function. Here, ∗ denotes the convolution operation, σ represents the activation function, which is rectified linear unit (ReLU), n denotes the total number of frames, W is the weight of the convolution kernel, and B is the bias. Finally, the output hidden state of each frame are concatenated to form the output feature map of the sequence, $F_{l}^{i}$, which subsequently serves as the input to the next layer within the current cascade block and also passed to the corresponding layer in the subsequent cascade block.

### 2.3 CRUNet‐MR

**FIGURE 1:**
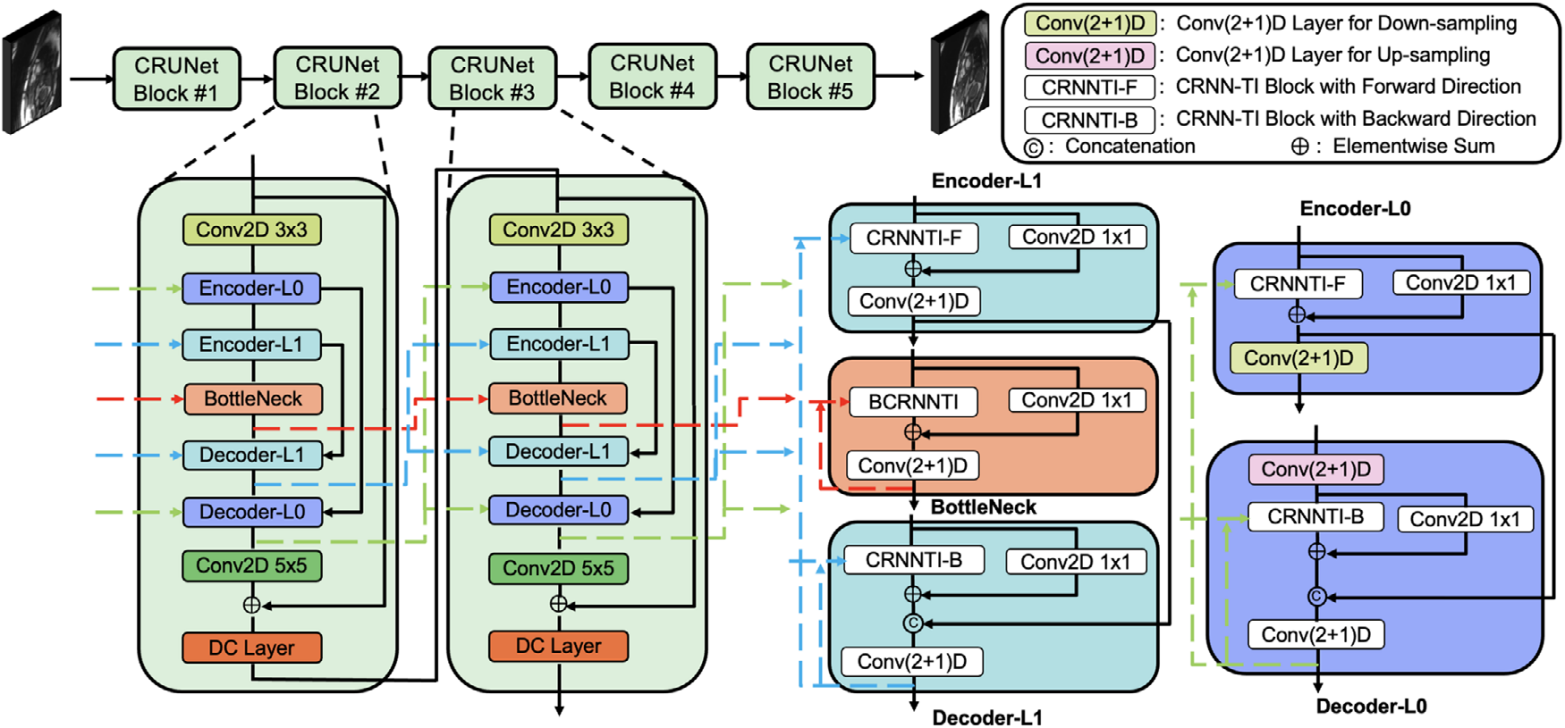
An overview of the CRUNet‐MR model structure: Inside each CRUNet block, green dashed lines represent information propagation at the first level across cascade blocks, blue dashed lines indicate information flow at the second level, and red dashed lines highlight information propagation through the bottleneck block across the cascade blocks.

Building on convolutional recurrent principles to better leverage the strong spatio‐temporal correlations in cine sequences, we developed a novel unrolled network model named CRUNet‐MR. The overall architecture, shown in Figure 1, consists of five CRUNet blocks. Each block combines convolutional recurrent units with a two‐level U‐Net structure.

For the cine MRI reconstruction task, given multi‐coil undersampled *k*‐space data, $y\in C^{T\times D_{C}\times D_{H}\times D_{W}}$, where $D_{C}$ is the number of coils, we initially apply an Inverse Fast Fourier Transform (IFFT) to obtain the image. Subsequently, a coil combination operation is performed, where each coil image is multiplied by its corresponding conjugated coil sensitivity map and summed along the coil dimension. This yields a combined single‐coil image $x_{0}\in R^{T\times D_{H}\times D_{W}\times 2}$, which is double‐channeled for real and imaginary parts. Then, the overall process with the CRUNet‐MR model unfolds as follows:

(12) $$x_{5}={\text{CRUNet-MR}}_{\theta }\left(H_{0},F_{0},x_{0},y,S,M\right),$$

where $H_{0}$ is the set of initial hidden states, $F_{0}$ denotes the set of feature maps from 0th iteration, both $H_{0}$ and $F_{0}$ are initialized as zero matrices, S represents the coil sensitivity map, and M is the undersampling mask. Taking $x_{0}$ as an initial input for CRUNet‐MR, the final output is $x_{5}\in R^{T\times D_{H}\times D_{W}\times 2}$. Additionally, y, S, and M contribute to the hard data consistency term of each cascade block, as defined by Equations (5) and (6). This term ensures that the information from the sampled *k*‐space region is accurately preserved throughout the reconstruction process. Furthermore, to enhance the stability of model training, we applied *z*‐score normalization at the beginning of each CRUNet block and un‐normalization at the end, ensuring the input is normalized before processing and returned to its original value range afterward.

To ensure consistent extraction of spatio‐temporal features within the cine sequence, the BCRNN‐TI operation is a suitable choice, as it employs recurrent operations across both the temporal dimension of the cine sequence and the cascade blocks. However, integrating the BCRNN‐TI operation directly into the U‐Net structure would highly increase resource consumption, making it hard to implement without sufficient GPU memory. To avoid this, inside each CRUNet block, we decomposed each BCRNN‐TI unit into two separate CRNN‐TI units with opposite propagation directions. One propagates information from front to back within the sequence, while the other operates in the reverse direction. These units are strategically positioned within the encoder and decoder at the same hierarchical level, effectively balancing computational efficiency with continuous spatio‐temporal feature extraction capabilities. Furthermore, additional Conv(2+1)D layers are incorporated to enhance the extraction of spatio‐temporal features from neighboring frames. In this context, a Conv2D layer is first applied to extract spatial features within each frame, followed by a Conv1D layer that facilitates interactions between adjacent frames, effectively capturing temporal dependencies. Considering the cyclical characteristics of cine MRI, where a strong correlation exists between the beginning and end of the cine sequence, we also incorporated temporal circular padding^21^ into all Conv(2+1)D layers. This involves wrapping the last frame before the first frame and placing the first frame after the last frame, enhancing the extraction of spatio‐temporal features at the both ends of the cine sequence.

Overall, the internal details of CRUNet block can be split into four parts in total. In the first part, two Conv2D layers at the beginning and end are used to adjust the number of channels and extract spatial features. The second part incorporates two CRNN‐TI blocks, operating in forward and backward directions, complemented by a Conv2D side branch to retain fine spatial details. Then, the outputs from the two CRNN‐TI blocks are merged via skip connections. This integration functions as an enhanced BCRNN‐TI operation, improving spatio‐temporal feature extraction by combining multi‐level features. The resulting feature map is then shared by both CRNN‐TI blocks in the subsequent cascade block, making them function as a cohesive unit. Furthermore, corresponding Conv(2+1)D layers are applied for downsampling and upsampling in this part. The third part, representing the second level of the CRUNet, follows a similar design to the first level but excludes downsampling and upsampling operations, avoiding spatial information loss. The final part serves as the bottleneck, consisting of a BCRNN‐TI block augmented with a Conv2D side branch, and concluding with a Conv(2+1)D layer.

Moreover, expanding the model's spatial receptive field should enhance the exploitation of spatial features. To investigate this impact, multiple dilation factors are incorporated into the convolutional operations within the CRUNet. The convolutional operations in the first two parts remain unchanged to maintain the extraction of detailed spatial features. After that, dilation factors are progressively increased by setting to 2 at the third part and 4 at the bottleneck. This adjustment primarily affects the convolution kernels within the CRNN‐TI blocks and the Conv2D component of the Conv(2+1)D layers, while the side branch retains standard convolutions to keep extracting some local details.

### 2.4 Loss function

Considering that both the *k*‐space domain and image domain are related to the performance of cine MRI reconstruction, we compose the loss function L by the *k*‐space domain $L_{kspace}$ as well as the image domain $L_{img}$.

(13) $$L=L_{kspace}+L_{img}.$$

We employ the mean squared error (MSE) loss to compute $L_{kspace}$:

(14) $$L_{kspace}=\lambda_{1}{∥y_{rec}-y_{gnd}∥}_{2}^{2},$$

where $y_{rec}$ denotes the reconstructed *k*‐space of cardiac cine MRI, obtained by applying a Fast Fourier Transform (FFT) to the reconstructed image, while $K_{gnd}$ represents the ground truth, which means fully‐sampled *k*‐space. Both are double‐channeled, with one channel representing the real part and the other representing the imaginary part. To prioritize image domain performance, we set $\lambda_{1}$ to 0.25.

Then, $L_{img}$ is the weighted sum of an L1 loss term, an MSE loss term and an SSIM loss term:

(15) $$\begin{array}{cc} L_{img} & =\lambda_{2}∥I_{rec}-I_{gnd}{∥}_{1}+\lambda_{3}{∥I_{rec}-I_{gnd}∥}_{2}^{2}\\ & \;+\lambda_{4}\left(1-\mathrm{SSIM}\left(I_{rec},I_{gnd}\right)\right). \end{array}$$

In this context, $I_{rec}$ refers to the reconstructed image series and $I_{gnd}$ represents the original ground‐truth image sequence, both of them are also double‐channeled. Here, both L1 loss and MSE loss focus on pixel‐level accuracy, each with distinct advantages for the reconstruction. We set $\lambda_{2}=\lambda_{3}=0.5$ to balance their contributions. Specifically, we compute the SSIM loss separately for the real and imaginary parts of the dual‐channel output, and then take their average to obtain the final SSIM loss. $\lambda_{4}$ is set to 1 to emphasize the structural information of reconstructed images.

## 3 EXPERIMENTS AND RESULTS

### 3.1 Dataset

#### 3.1.1 CMRxRecon2023 dataset

We used the CMRxRecon2023^22^ dataset for evaluating model's performance, which was acquired with a 3T MRI scanner (MAGNETOM Vida, Siemens Healthineers, Germany) from 300 healthy volunteers (160 females and 140 males; mean age: 26±5 years) between June 2022 and March 2023. Cardiac cine acquisitions were performed using a TrueFISP readout and a retrospective ECG‐gated segmented approach, with *k*‐space sampled over multiple cardiac cycles along the phase‐encoding direction. The cine images included short‐axis (SAX), two‐chamber (2CH), three‐chamber (3CH), and four‐chamber (4CH) long‐axis (LAX) views. For SAX view, 5–14 slices were typically collected, whereas only a single slice was acquired for the other views. Each cardiac cycle was divided into 12–25 phases with a temporal resolution ∼50 ms according to the heart rate. Typical scan parameters are: spatial resolution of 1.5×1.5 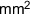, slice thickness of 8.0 mm, repetition time (TR) of 3.6 ms, echo time (TE) of 1.6 ms, and field‐of‐view (FOV) of 340 × 300 ${\mathrm{mm}}^{2}$ (LAX) or 340 × 340 ${\mathrm{mm}}^{2}$ (SAX). Signal acquisition was performed during breath‐holds (2 for LAX, 11 for SAX), automatically optimized based on acquisition size, heart rate, and slices, with a maximum duration of 12 s. Processing steps included coil compression to 10 virtual coils, the filling of partial Fourier data using the POCS algorithm,^23^ applying GRAPPA^24^ to under‐sampled *k*‐space (*R* = 3) to acquire full k‐space data, and exclusion of poor‐quality images based on expert visual assessment.

The dataset features acceleration factors of 4, 8, and 10, implemented with uniform Cartesian sampling outside the Auto‐Calibration Signal (ACS) area (covering the central 24 lines). The undersampling mask remains consistent across all frames. Notably, the ACS area reduces effective acceleration to about 3 for *R* = 4 and 5 for *R *= 8 and *R* = 10. Given the similar factual acceleration for *R* = 8 and *R* = 10 and the purpose of further exploring the model's potential at higher acceleration, an additional factor of 24 was introduced, using the same sampling strategy with a gap of 24 outside the ACS area, resulting in an effective acceleration of approximately 8. The dataset contains 12 frames per case and is available in both single‐coil and multi‐coil formats. However, this work focuses solely on multi‐coil data, reflecting the typical clinical acquisition method. Additionally, the dataset includes 120 subjects for training, 60 for validation, and 120 for testing, with this split preserved throughout training.

#### 3.1.2 LUMC in‐house dataset

We additionally collected an LUMC in‐house dataset to evaluate the CRUNet‐MR model. The dataset consists of 46 patients acquired on a 3T MRI scanner (Ingenia, Philips, Best, The Netherlands) with an acceleration factor of 4. The study has been approved for research purposes by the institutional review board. For each subject, 14 short‐axis (SAX) slices were acquired. A balanced turbo field echo (B‐TFE) sequence was used for cardiac cine acquisition. The scan parameters are as follows: slice thickness of 8.0 mm; repetition time (TR) of 3.14 ms; echo time (TE) of 1.57 ms; in‐plane spatial resolution of 1×1 ${\mathrm{mm}}^{2}$; FOV of 378×706 ${\mathrm{mm}}^{2}$; flip angle of $45^{\circ }$. The scans were acquired over 15 breath‐holds, each lasting approximately 6 s. To note, the LUMC data employed the same *k*‐space sampling pattern across all frames without an ACS region. During fine‐tuning of the CRUNet‐MR model, 36 subjects were used for training and the remaining 10 subjects for testing.

### 3.2 Implementation details

To comprehensively evaluate CRUNet‐MR, we used four metrics: Peak Signal‐to‐Noise Ratio (PSNR), Structural Similarity (SSIM), Deep Image Structure and Texture Similarity (DISTS)^25^, and the Haar Wavelet‐based Perceptual Similarity Index (HaarPSI).^26^ PSNR and SSIM standard for reconstruction tasks, while DISTS and HaarPSI assess perceptual performance, better correlating with radiological assessments^27^ and potentially reflecting clinical relevance. Statistical significance for pairwise method comparisons under each metric was assessed using the Wilcoxon signed‐rank test (p=0.05), with Bonferroni correction applied to control for multiple method comparisons. Adjusted *p*‐value thresholds were set based on the number of comparisons: p=0.0083 for Table 1, p=0.0167 for Tables 3 and 4, and p=0.025 for Tables 5 and 6.

**TABLE 1:** Comparison of CRUNet‐MR with benchmarks on the CMRxRecon2023 test set.

View	*R*	Models	PSNR ↑	SSIM ↑	DISTS ↑	HaarPSI ↑
Multi‐Coil LAX	4×	GRAPPA	42.78±2.37 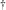	0.962±0.016 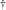	0.933±0.013 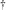	0.955±0.017 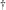
		L+S	34.83±3.73 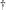	0.893±0.065 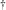	0.875±0.038 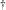	0.758±0.115 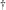
		CineNet	38.26±2.87 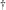	0.951±0.024 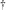	0.904±0.022 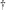	0.865±0.057 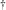
		3D UNet‐MR	43.89±2.73 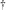	0.981±0.010 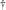	0.950±0.015 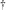	0.950±0.030 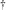
		CRNN‐MRI	44.17±2.77 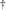	0.983±0.009 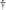	0.954±0.014 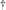	0.953±0.029 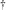
		PromptMR	41.54±4.72 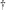	0.982±0.017 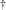	0.953±0.015 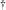	0.945±0.054 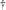
		CRUNet‐MR	45.74±2.96	0.986±0.009	0.962±0.014	0.966±0.028
	8×	GRAPPA	32.93±2.08 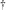	0.870±0.029 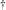	0.851±0.014 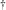	0.725±0.056 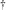
		L+S	29.38±2.80 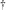	0.806±0.068 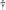	0.812±0.030 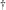	0.575±0.096 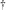
		CineNet	33.33±2.63 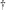	0.906±0.038 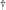	0.851±0.019 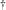	0.731±0.079 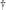
		3D UNet‐MR	38.16±2.25 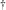	0.957±0.016 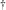	0.907±0.014 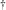	0.865±0.044 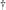
		CRNN‐MRI	39.33±2.42 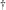	0.965±0.013 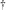	0.916±0.013 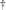	0.893±0.044 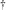
		PromptMR	38.92±3.78 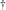	0.970±0.021 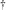	0.924±0.015 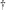	0.903±0.063 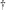
		CRUNet‐MR	40.97±2.83	0.971±0.014	0.929±0.015	0.918±0.052
	10×	GRAPPA	32.12±2.37 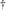	0.867±0.037 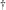	0.848±0.017 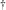	0.701±0.075 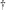
		L+S	28.98±2.81 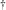	0.798±0.070 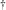	0.806±0.031 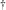	0.567±0.102 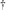
		CineNet	32.60±2.55 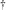	0.898±0.039 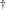	0.842±0.020 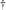	0.709±0.079 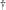
		3D UNet‐MR	37.20±2.15 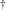	0.953±0.015 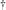	0.899±0.013 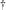	0.847±0.047 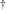
		CRNN‐MRI	38.28±2.23 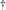	0.959±0.014 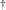	0.907±0.013 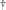	0.874±0.047 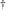
		PromptMR	38.19±3.63 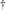	0.967±0.021 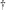	0.916±0.015 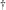	0.891±0.068 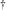
		CRUNet‐MR	39.84±2.84	0.966±0.016	0.920±0.015	0.903±0.058
	24×	GRAPPA	29.53±2.67 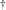	0.847±0.042 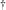	0.822±0.020 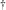	0.609±0.087 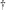
		L+S	27.62±2.62 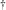	0.789±0.067 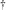	0.792±0.028 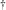	0.514±0.092 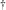
		CineNet	31.02±2.33 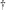	0.884±0.035 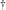	0.821±0.019 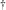	0.652±0.080 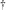
		3D UNet‐MR	33.39±1.98 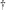	0.924±0.021 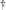	0.857±0.014 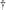	0.742±0.057 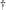
		CRNN‐MRI	34.20±2.00 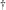	0.932±0.019 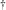	0.867±0.013 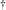	0.773±0.055 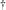
		PromptMR	34.80±2.67 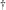	0.948±0.023 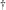	0.886±0.014 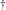	0.814±0.069
		CRUNet‐MR	35.51±2.32	0.943±0.020	0.888±0.014	0.818±0.063
Multi‐Coil SAX	4×	GRAPPA	42.40±2.27 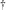	0.958±0.015 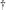	0.927±0.012 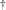	0.953±0.018 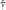
		L+S	35.65±3.81 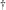	0.906±0.058 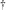	0.878±0.039 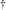	0.779±0.113 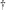
		CineNet	39.69±3.09 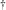	0.959±0.023 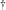	0.912±0.024 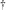	0.882±0.056 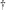
		3D UNet‐MR	44.02±2.90 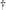	0.982±0.010 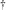	0.951±0.016 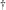	0.947±0.032 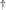
		CRNN‐MRI	44.48±2.95 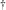	0.983±0.009 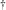	0.954±0.014 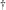	0.952±0.030 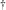
		PromptMR	40.64±5.05 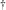	0.981±0.020 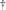	0.953±0.018 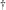	0.937±0.062 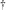
		CRUNet‐MR	46.17±3.10	0.987±0.008	0.964±0.013	0.967±0.025
	8×	GRAPPA	34.18±1.96 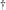	0.885±0.028 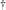	0.852±0.016 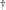	0.756±0.051 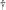
		L+S	30.53±2.72 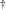	0.829±0.062 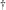	0.817±0.032 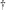	0.607±0.094 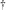
		CineNet	34.90±2.83 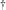	0.920±0.037 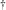	0.860±0.022 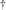	0.760±0.078 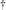
		3D UNet‐MR	39.80±2.42 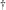	0.964±0.013 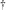	0.916±0.012 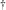	0.891±0.043 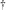
		CRNN‐MRI	39.97±2.49 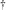	0.965±0.014 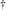	0.916±0.013 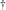	0.892±0.045 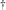
		PromptMR	38.97±4.27 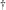	0.972±0.022 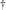	0.924±0.017 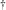	0.904±0.067 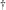
		CRUNet‐MR	41.75±2.86	0.973±0.013	0.932±0.013	0.923±0.045
	10×	GRAPPA	32.71±2.01 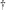	0.872±0.030 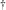	0.840±0.016 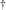	0.708±0.046 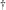
		L+S	29.56±2.58 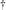	0.814±0.062 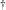	0.807±0.031 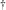	0.572±0.087 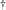
		CineNet	34.05±2.91 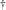	0.912±0.041 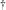	0.851±0.022 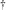	0.734±0.081 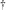
		3D UNet‐MR	38.55±2.40 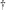	0.957±0.015 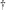	0.905±0.012 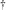	0.864±0.048 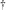
		CRNN‐MRI	38.85±2.46 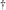	0.959±0.015 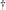	0.906±0.013 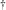	0.870±0.048 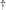
		PromptMR	38.32±4.04 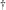	0.968±0.022 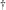	0.914±0.016 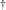	0.889±0.069 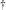
		CRUNet‐MR	40.64±2.90	0.968±0.015	0.923±0.013	0.906±0.052
	24×	GRAPPA	30.48±2.29 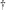	0.862±0.036 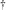	0.825±0.019 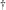	0.622±0.066 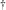
		L+S	28.40±2.35 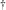	0.807±0.060 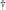	0.797±0.030 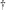	0.527±0.080 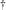
		CineNet	32.08±2.42 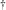	0.897±0.038 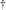	0.829±0.019 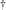	0.667±0.074 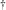
		3D UNet‐MR	34.79±1.93 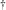	0.932±0.019 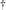	0.870±0.012 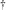	0.766±0.049 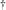
		CRNN‐MRI	35.03±2.00 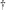	0.933±0.019 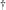	0.870±0.013 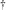	0.773±0.050 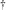
		PromptMR	35.38±2.95 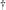	0.950±0.024 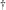	0.887±0.015 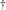	0.815±0.066 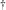
		CRUNet‐MR	36.46±2.14	0.945±0.018	0.892±0.012	0.819±0.052

*Note*: Best results in **bold**. †: p<0.0083 (Bonferroni correction with Wilcoxon signed‐rank test, pairwise comparison against CRUNet‐MR).

Abbreviations: DISTS, deep image structure and texture similarity; HaarPSI, Haar wavelet‐based perceptual similarity index; PSNR, peak signal‐to‐noise ratio; SSIM, structural similarity.

Before training, we normalized the multi‐coil *k*‐space data by transforming it into the image domain using an Inverse Fast Fourier Transform (IFFT). We then scaled each data sample by dividing it by its maximum absolute value. Finally, the normalized image data was transformed back to the original *k*‐space format using a Fast Fourier Transform (FFT).

We compared CRUNet‐MR with two traditional reconstruction methods and four related deep‐learning‐based benchmark models, including GRAPPA,^24^ L+S,^10^ CineNet,^13^ CRNN‐MRI,^15^ 3D UNet‐MR, and PromptMR.^18^ All methods were implemented in PyTorch 1.11.0 and trained on an NVIDIA RTX A6000 GPU (48 GB memory), while inference speed was evaluated on an NVIDIA Quadro RTX 6000 GPU (24 GB memory). Deep learning models used a cascade number of 5, with consistent hyper‐parameters across all experiments, except for PromptMR. Considering PromptMR is designed to handle both cine data and T1/T2 mapping data and our focus is solely on cine data, we retrained it using only cine data. As mentioned earlier, PromptMR reconstructs one frame at a time using 5 frames as input, resulting in 12 times more update iterations per epoch compared to methods that reconstruct the entire sequence at once. Given this difference, the cosine scheduler in our settings is not well‐suited for PromptMR's training. Therefore, we retained PromptMR's original configuration to ensure its superior performance. The total number of training epochs is set to 12, with the learning rate fixed $2\times 10^{-4}$ for the first 11 epochs and $2\times 10^{-5}$ for the last epoch. The AdamW optimizer was used with a weight decay of 0.01. Then, for the hyper‐parameter settings of other deep learning methods, we used AdamW as the optimizer with an initial learning rate of $3\times 10^{-4}$ and a weight decay of 0.01. The batch size is set to 1. To achieve dynamic learning rate adjustments and enhance training efficiency, we applied a cosine‐annealing scheduler with 10 warm‐up epochs and a minimal learning rate of $1\times 10^{-4}$. We set the total training epochs to 144 for matching the number of update iterations with the PromptMR model, ensuring that all models converge effectively. Code is available at https://github.com/dong845/CRUNet‐MR/tree/main.

In the fine‐tuning experiment of CRUNet‐MR on the LUMC in‐house dataset, we used an NVIDIA RTX A6000 GPU (48 GB memory). Training was performed for 32 epochs with a batch size of 1. We employed the AdamW optimizer with an initial learning rate of $1\times 10^{-4}$ and a weight decay of 0.01. A cosine‐annealing learning rate scheduler was applied with 5 warm‐up epochs and a minimum learning rate of $5\times 10^{-5}$. Notably, due to the considerably larger image size of the in‐house dataset compared to public datasets, current available GPU memory could not accommodate training with all frames simultaneously. Therefore, we adopted the strategy from PromptMR, where five consecutive frames were used as input to reconstruct the middle frame.

### 3.3 Quantitative and qualitative comparison with baselines

To comprehensively evaluate CRUNet‐MR, we compared it with two traditional methods and four deep learning models, including well‐established baseline models in the field as well as the state‐of‐the‐art model from the CMRxRecon2023 dataset: (1) GRAPPA:^24^ GRAPPA uses fully sampled auto‐calibration signal (ACS) region to learn convolutional weights, which are then applied to synthesize missing *k*‐space data from multi‐coil signals; (2) L+S:^10^ L+S (Low‐rank plus Sparse) models the image sequence as the sum of a low‐rank component, capturing temporally correlated background information, and a sparse component, representing dynamic or transient features; (3) CineNet:^13^ CineNet employs a lightweight CNN‐based U‐Net to reduce undersampling artifacts and noise in sparse domains by applying a Fourier Transform along the temporal dimension of the image, reshaping it into the xt and yt domains for targeted processing; (4) CRNN‐MRI:^15^ CRNN‐MRI adopts a straightforward streamlined structure, integrating convolutional recurrent operations across the temporal dimension of the sequence and cascade blocks, aiming to leverage spatio‐temporal features throughout the entire sequence; (5) 3D UNet‐MR: 3D UNet‐MR is a modified unrolled U‐Net model derived from CRUNet‐MR by replacing all CRNN‐TI blocks with Conv3D blocks while retaining the overall structure. This can also serve as a baseline to evaluate the contribution of convolutional recurrent units to performance; (6) PromptMR:^18^ PromptMR achieves the best performance on the CMRxRecon2023 dataset, reconstructing the center frame from five input frames. It applies channel attention across combined temporal‐channel dimensions for global spatio‐temporal modeling, and introduces a learnable prompt to adapt to both cine and T1/T2 mapping data while enriching spatial information. LAX, long axis; SAX, short axis.

Table 1 presents the overall performance of the methods across two views and four acceleration factors using the testing set from the CMRxRecon2023 dataset, comprising 211 slices of LAX and 879 slices of SAX in total. Overall, deep learning models significantly outperformed the two traditional methods. GRAPPA performed reasonably well at an acceleration factor of 4 due to the presence of ACS lines, but showed substantial performance degradation at higher factors. L+S struggled across all acceleration factors, likely due to the use of a fixed sampling mask across frames, which limits its ability to exploit temporal information. Among deep learning models, CineNet exhibited suboptimal reconstruction capabilities across both views, and 3D UNet‐MR underperformed compared to CRNN‐MRI. Likewise, CRUNet‐MR demonstrated a substantial improvement over its base model, CRNN‐MRI. Although PromptMR showed much better overall performance than the other benchmark models, CRUNet‐MR still demonstrated superior performance at lower acceleration factors (*R* = 4 and *R* = 8), while outperforming PromptMR at higher acceleration factors (*R *= 10 and *R* = 24) in certain metrics, showing better overall performance.

**FIGURE 2:**
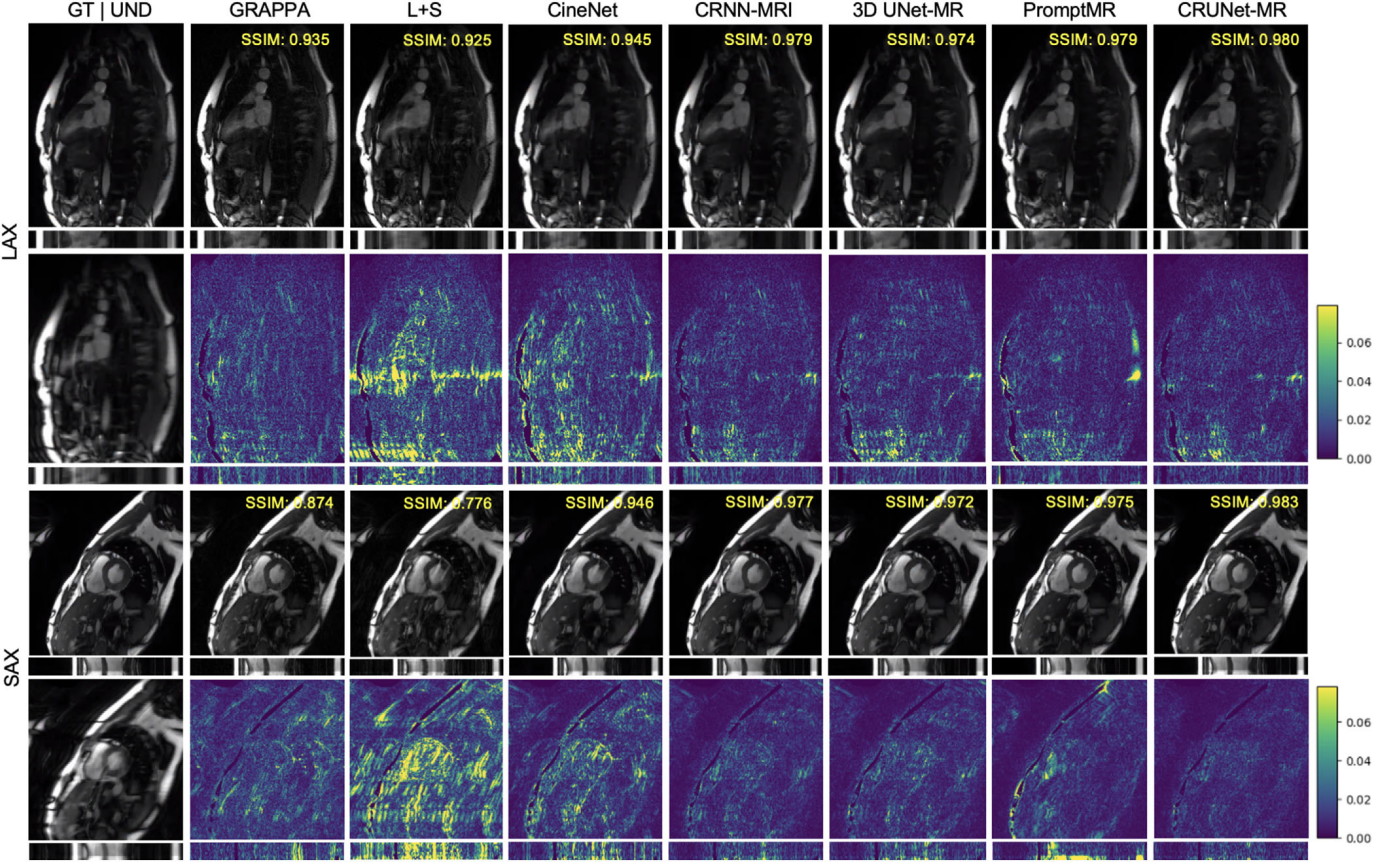
Visualization of models' results on LAX and SAX views of the CMRxRecon2023 test data at *R* = 4. ”GT” stands for the ground truth, and ”UND” denotes the corresponding undersampled image. LAX, long axis; SAX, short axis.

**FIGURE 3:**
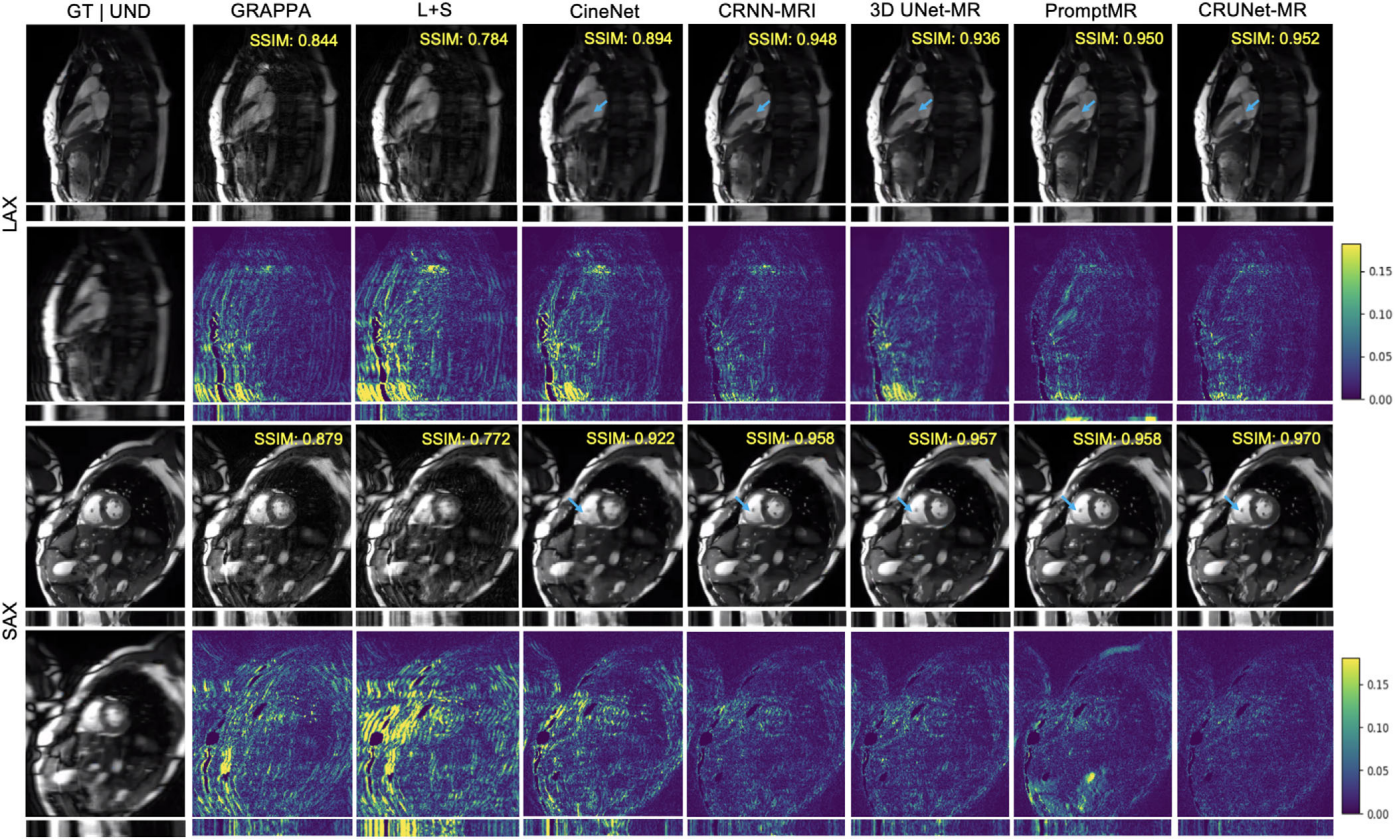
Visualization of models' results on LAX and SAX views of the CMRxRecon2023 test data at *R* = 8, with blue arrows highlighting areas for comparison.

Figures 2 and 3 present the visualizations of reconstructed results and corresponding error maps for two test samples from different views at acceleration factors of 4 and 8, respectively, offering further insights into the models' performance. To note, we cropped the top and bottom quarters of each frame to remove the black part and focus on showing the detailed heart region. The bottom section under each image displays the result along the central line across the temporal dimension, while the SSIM value in the top‐right corner reflects the model's performance over the entire cropped cine sequence. From these figures, it is clear that both two traditional methods and CineNet struggled with aliasing artifacts, although CineNet was less affected. CRNN‐MRI and 3D UNet‐MR achieved better reconstruction quality, but they still failed to capture fine details in intricate regions. For instance, in LAX views at acceleration factors of 4 and 8, some boundaries inside the cardiac region become heavily blurred and even vanish as the acceleration factor increases. In contrast, both PromptMR and CRUNet‐MR effectively recovered most structural cardiac details and highly reduced artifacts, despite PromptMR showing a lower SSIM value. To note, the error maps of PromptMR in the cardiac region revealed poorer performance compared to CRUNet‐MR, highlighting its weaker pixel‐level accuracy. Additionally, some detailed cardiac features reconstructed by PromptMR appeared less sharp, which is indicated by blue arrows in Figure 3, emphasizing CRUNet‐MR's better ability to reconstruct the dynamic cardiac region.

### 3.4 Dynamic region analysis

**FIGURE 4:**
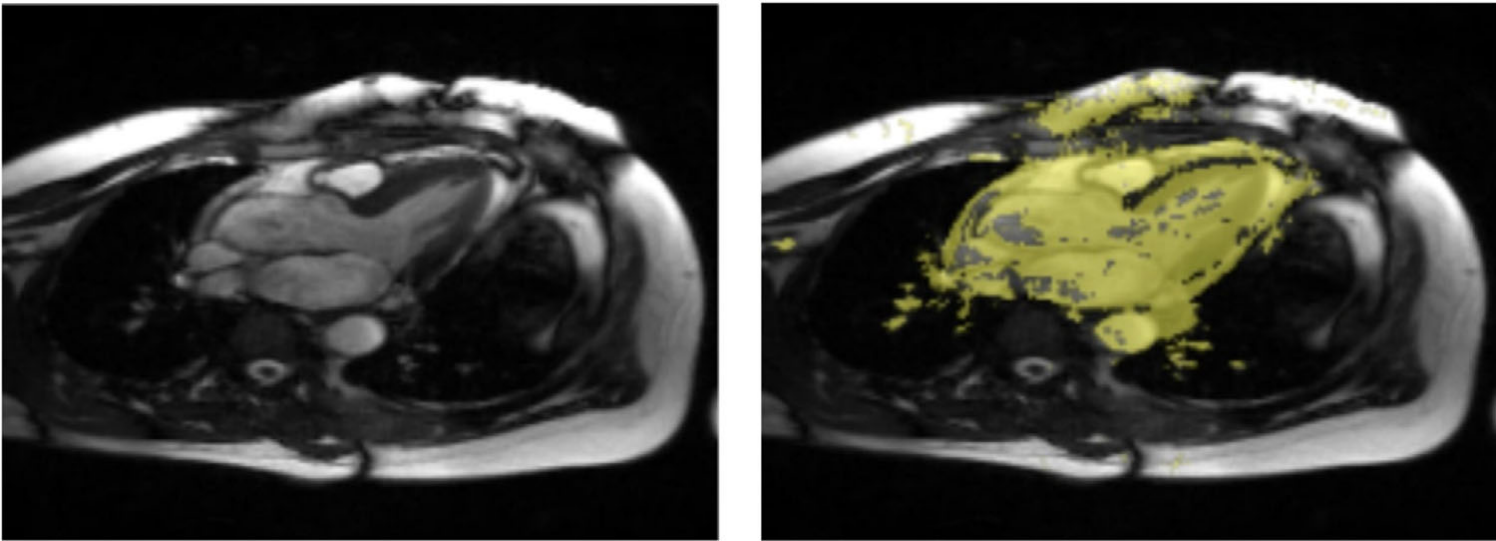
The dynamic region across the cardiac cine sequence, as represented by the generated mask.

In cardiac cine MRI, the highly dynamic region, characterized by rapid motion and complex patterns, exposes the greatest challenge for reconstruction. Accurate performance in this region reflects a model's ability to exploit spatio‐temporal features. Due to the substantial displacement in the dynamic region, pixel values change drastically across the sequence, leading to a high standard deviation along temporal dimension. To this end, we computed the standard deviation of ground‐truth intensities along the temporal axis and set the 90th percentile as a threshold to automatically identify the highly dynamic region in the cine sequence. As illustrated in Figure 4, this approach effectively shows that dynamic region primarily corresponds to the heart region due to its motion, demonstrating that the chosen threshold reliably isolates dynamic areas in cardiac cine MRI.

**FIGURE 5:**
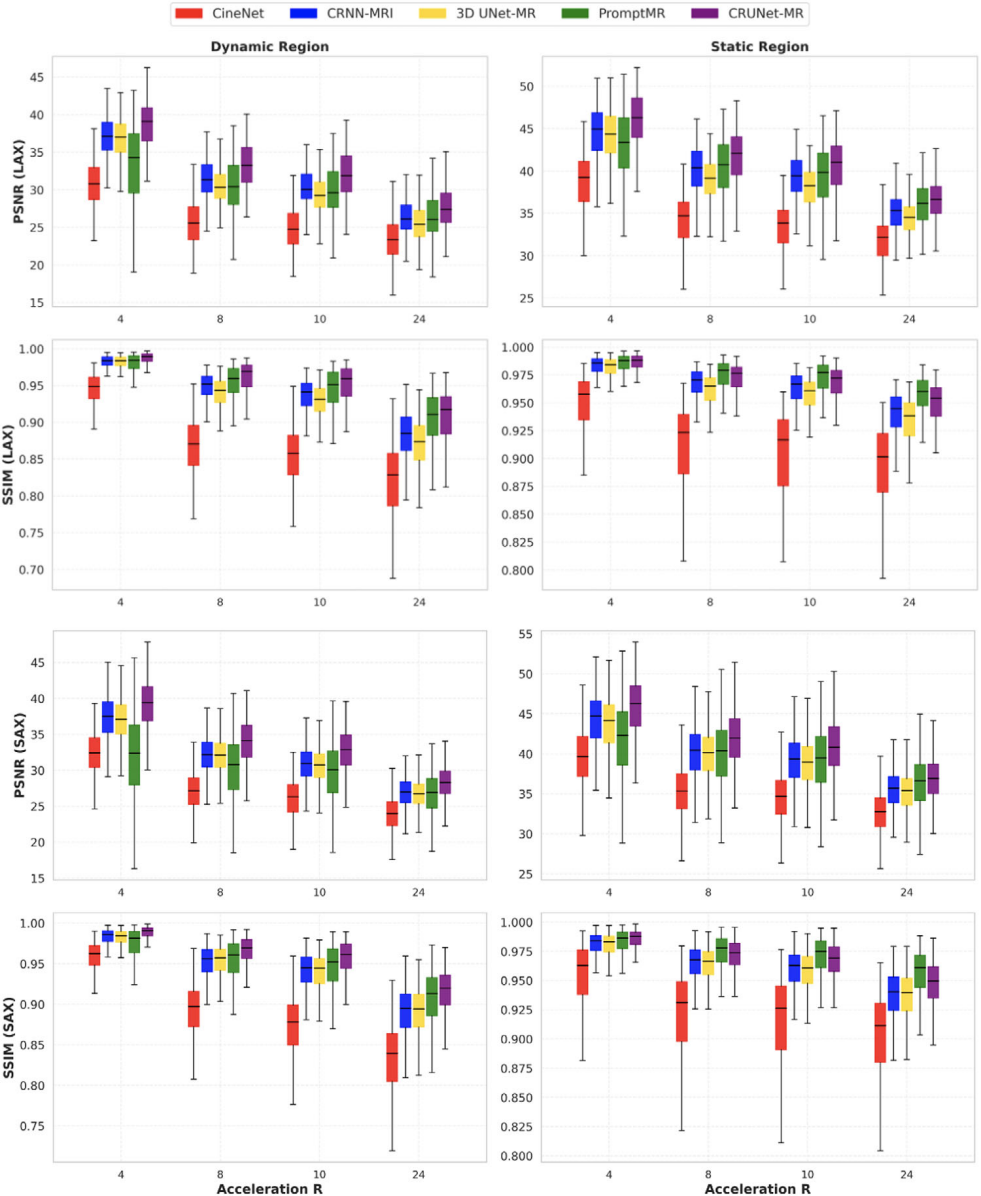
Dynamic analysis of the models on the CMRxRecon2023 test data, with the upper part representing LAX results and the bottom part showing SAX results. LAX, long axis; SAX, short axis.

By defining the dynamic region in this manner, the entire cine sequence can be divided into two segments: the dynamic region and the remaining area with small motion, which we refer to as the “static region” in contrast to the dynamic region. PSNR and SSIM were selected as the primary evaluation metrics for this dynamic analysis, as DISTS and HaarPSI are not suitable for point sets. We then calculated the PSNR and SSIM values of each region separately to more comprehensively assess the models' effectiveness in exploiting spatio‐temporal features. Figure 5 provides an overview of the models' performance across these two regions. Overall, CRUNet‐MR achieved the highest PSNR values across both views and regions and the highest SSIM in the dynamic region. However, in the static region, CRUNet‐MR exhibited lower SSIM compared to PromptMR, except at an acceleration factor of 4.

### 3.5 Analysis of parameter count and inference time

While the above tables and figures provide comprehensive quantitative and qualitative evaluations about reconstruction performance, it is also important to assess additional aspects of models, including parameter count and average inference time for each slice sequence including all phases, as they are critical factors in practical reconstruction applications. Accordingly, we calculated the parameter count for each deep learning method and measured their inference time on the CMRxRecon2023 test set under identical hardware conditions. Given the size differences between LAX and SAX views, inference times are reported separately for each. Although all models were tested across four acceleration factors, the variation in inference time was negligible, so we report the average inference time for each view. The results for all deep learning models are summarized in Table 2.

**TABLE 2:** Quantitative analysis of parameter count and per‐slice inference time for each deep learning model across the two views of the CMRxRecon2023 test data.

Models	Parameter count (MB) ↓	LAX inference time (seconds) ↓	SAX inference time (seconds) ↓
CineNet	**0.65**	**0.06**	**0.08**
3D UNet‐MR	4.00	0.07	0.44
CRNN‐MRI	1.49	0.74	1.03
PromptMR	30.50	3.50	4.73
CRUNet‐MR	4.79	1.45	2.00

*Note*: Best results are highlighted in **bold**. Abbreviations: LAX, long axis; SAX, short axis.

In general, CineNet is the most lightweight deep learning method, whereas PromptMR has the largest number of parameters due to its use of a channel attention mechanism and deeper U‐Net structure. CRUNet‐MR has more parameters than both 3D UNet‐MR and CRNN‐MRI, because it combines a U‐Net architecture with convolutional recurrent operations. In terms of inference time, all methods remain within an acceptable range. CineNet is the fastest, consistent with its small parameter count, while PromptMR is the slowest since it reconstructs frames sequentially. CRUNet‐MR is slower than both CRNN‐MRI and 3D UNet‐MR, and despite CRNN‐MRI having fewer parameters than 3D UNet‐MR, its recurrent operations introduce additional latency due to inter‐frame information propagation.

### 3.6 Ablation study

#### 3.6.1 Model components

In this section, we conducted ablation studies to evaluate the impact of some key components within the model design. The study examines three key components: skip connections (removing all side branches adjacent to the CRNN‐TI blocks), temporal padding in Conv(2+1)D layers (disabling temporal padding), and CRNN‐TI blocks (eliminating recurrent operations spanning cascade blocks, focusing solely on recurrent operations within the cine sequence). All experiments were conducted using the same hyperparameter settings as mentioned before, based on the LAX view dataset with an acceleration factor of 8. The results are shown in the Table 3. In general, the limited data volume of the LAX view dataset prevents the impacts of some components from being clearly distinct. Among these three components, the CRNN‐TI operation had the largest impact, as it was the primary component for extracting spatio‐temporal features across the sequence. Removing information propagation over the cascade blocks led to a notable loss of critical features from earlier blocks, leading to a sharp decline in performance. The other two components had smaller but still positive effects, underscoring the potential benefits of incorporating these operations.

**TABLE 3:** Ablation study of model components on LAX view of the CMRxRecon2023 test data at *R *= 8.

Skip connection	Temporal padding	CRNN‐TI	PSNR ↑	SSIM ↑
✓	✓	✗	40.35±2.80 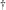	0.967±0.015 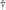
✗	✓	✓	40.81±2.76 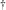	0.970±0.014 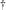
✓	✗	✓	40.90±2.80	0.970±0.014 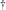
✓	✓	✓	40.97±2.83	0.971±0.014

*Note*: Best results are in **bold**. †: p<0.0167 (Bonferroni correction with Wilcoxon signed‐rank test, pairwise comparison against CRUNet‐MR with all model components).

Abbreviations: PSNR, peak signal‐to‐noise ratio; SSIM, structural similarity.

#### 3.6.2 Loss function terms

**FIGURE 6:**
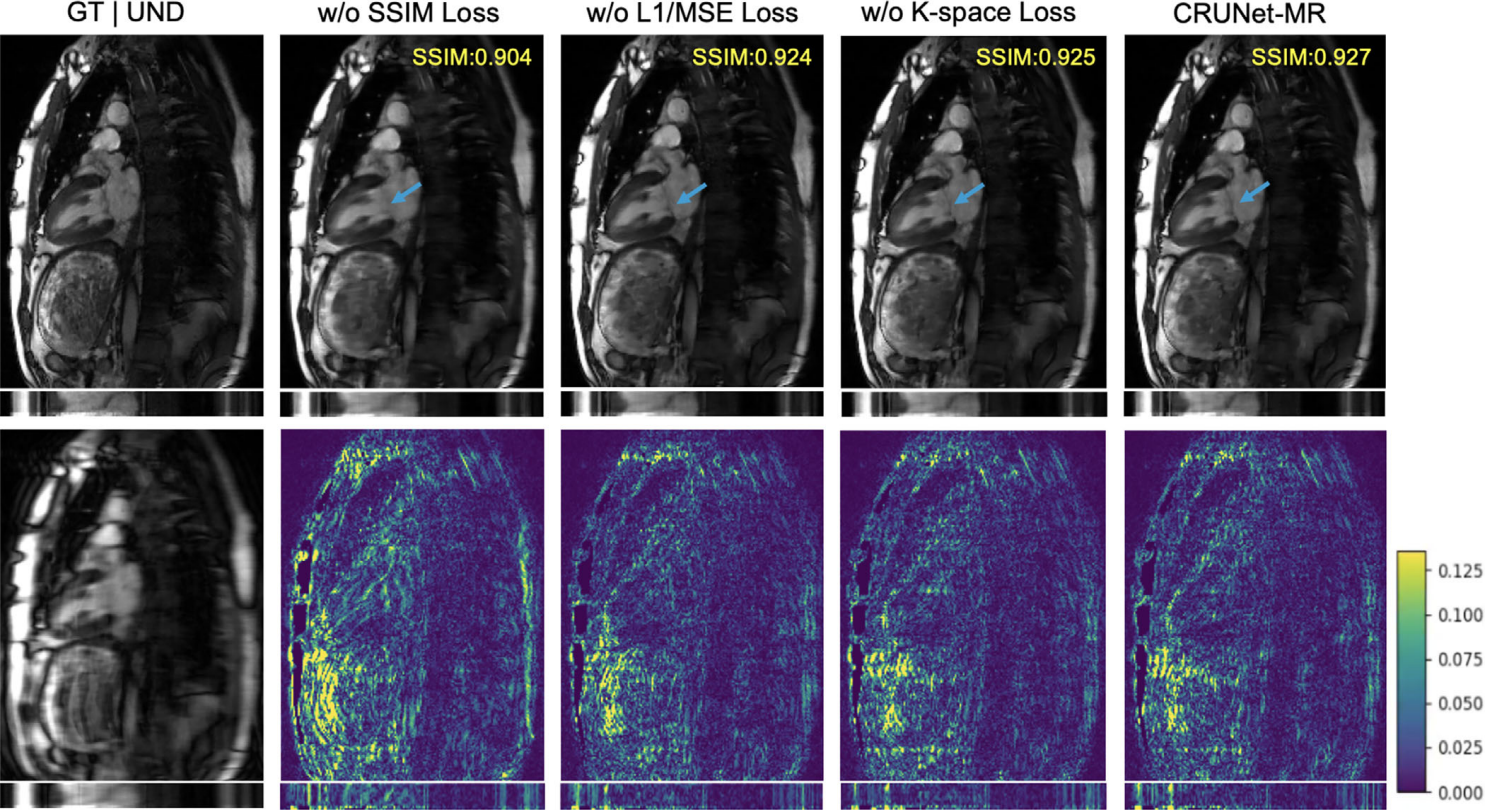
Visualization of the CRUNet‐MR model outputs without each loss term on the LAX view of the CMRxRecon2023 test data at *R* = 8, with blue arrows highlighting the differences and the SSIM value in the top‐right corner indicating performance over the cropped cine sequence. LAX, long axis; SSIM, structural similarity.

During training, the loss function plays a crucial role in guiding model optimization. As described earlier, our loss function consists of three components: an MSE loss in the *k*‐space domain, a combined L1/MSE loss in the image domain, and an SSIM loss in the image domain. To assess the contribution of each component and determine their necessity in training CRUNet‐MR, we conducted a corresponding ablation study by systematically removing each term from the overall loss function and evaluating the resulting reconstruction performance. The overall performance is summarized in Table 4 and Figure 6.

**TABLE 4:** Ablation study of loss function terms on LAX view of the CMRxRecon2023 test data at *R* = 8.

*K*‐space MSE loss	Image L1/MSE loss	Image SSIM loss	PSNR ↑	SSIM ↑
✓	✓	✗	36.98±3.39 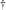	0.959±0.017 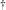
✗	✓	✓	40.87±2.83	0.970±0.014
✓	✗	✓	40.83±2.85 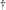	0.970±0.014 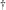
✓	✓	✓	40.97±2.83	0.971±0.014

*Note*: Best results are in **bold**. †: p<0.0167 (Bonferroni correction with Wilcoxon signed‐rank test, pairwise comparison against CRUNet‐MR with all loss terms).

Abbreviations: PSNR, peak signal‐to‐noise ratio; SSIM, structural similarity.

The results clearly show that the SSIM loss in the image domain is crucial for cine MRI reconstruction, as its removal lead to a substantial drop in performance and visibly blurred fine structures. In contrast, the MSE loss in the *k*‐space domain and the combined L1/MSE loss in the image domain have a smaller impact: slightly reduces quantitative scores while yielding visually similar reconstructions. Notably, omitting the *k*‐space loss does not cause statistically significant changes in the metrics, suggesting it could be excluded without materially affecting performance. However, as shown in Figure 6, incorporating the *k*‐space loss alongside the combined L1/MSE loss still enhances subtle details and sharpens features, as indicated by the blue arrow.

#### 3.6.3 Dilation factors

As previously mentioned, we aimed to investigate the relationship between the model's receptive field and reconstruction performance by introducing multiple dilation factors in the U‐Net structure. Therefore, we conducted a related ablation study by incorporating various dilation factor combinations into CRUNet‐MR.

Each CRUNet block adopted a two‐level U‐Net structure with a bottleneck, where we assigned specific dilation factor to each level. We experimented with three dilation factor combinations: (1, 1, 1), (1, 2, 4), and (1, 3, 5). Here, the first one served as a baseline using standard convolutions without dilation. As shown in Table 5, the (1, 2, 4) configuration yielded the best overall performance for acceleration factors of 4, 8, and 10, although it slightly underperformed (1, 1, 1) in the LAX view at factors 8 and 10 on certain metrics, likely due to the smaller number of training samples in that view, which also reduced the clarity of statistical significance. For an acceleration factor of 24, the (1, 3, 5) combination proved most effective, slightly outperforming (1, 2, 4) and substantially surpassing (1, 1, 1).

**TABLE 5:** CRUNet‐MR results with different dilation factors on LAX and SAX views of the CMRxRecon2023 test data.

View	R	Dilations	PSNR ↑	SSIM ↑	DISTS ↑	HaarPSI ↑
Multi‐Coil LAX	4×	(1, 1, 1)	45.52±2.84 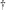	0.986±0.008 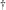	0.961±0.013 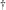	0.965±0.026 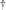
		(1, 3, 5)	45.72±3.04	0.986±0.009	0.962±0.015	0.965±0.029
		(1, 2, 4)	45.74±2.96	0.986±0.009	0.962±0.014	0.966±0.028
	8×	(1, 1, 1)	40.96±2.70	0.971±0.013	0.928±0.014 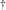	0.921±0.047
		(1, 3, 5)	40.95±2.90	0.970±0.015 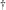	0.928±0.016 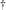	0.917±0.054
		(1, 2, 4)	40.97±2.83	0.971±0.014	0.929±0.015	0.918±0.052
	10×	(1, 1, 1)	39.85±2.68	0.967±0.015 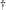	0.920±0.013 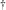	0.906±0.054
		(1, 3, 5)	39.73±2.90 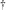	0.965±0.016 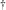	0.919±0.016 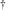	0.901±0.060 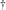
		(1, 2, 4)	39.84±2.84	0.966±0.016	0.920±0.015	0.903±0.058
	24×	(1, 1, 1)	35.02±2.05 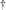	0.938±0.018 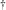	0.882±0.012 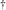	0.801±0.053 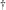
		(1, 3, 5)	35.51±2.42	0.943±0.021 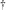	0.888±0.016	0.820±0.068 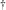
		(1, 2, 4)	35.51±2.32	0.943±0.020	0.888±0.014	0.818±0.063
Multi‐Coil SAX	4×	(1, 1, 1)	45.93±3.05 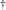	0.987±0.008 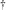	0.963±0.013 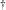	0.966±0.025 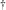
		(1, 3, 5)	46.07±3.17 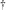	0.987±0.008 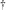	0.964±0.013	0.966±0.026 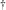
		(1, 2, 4)	46.17±3.10	0.987±0.008	0.964±0.013	0.967±0.025
	8×	(1, 1, 1)	41.61±2.77 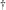	0.972±0.013 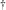	0.930±0.013 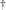	0.922±0.044 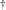
		(1, 3, 5)	41.65±2.95 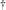	0.972±0.013 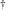	0.932±0.014	0.921±0.048 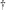
		(1, 2, 4)	41.75±2.86	0.973±0.013	0.932±0.013	0.923±0.045
	10×	(1, 1, 1)	40.59±2.79 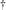	0.967±0.014 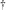	0.921±0.013 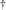	0.906±0.050 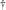
		(1, 3, 5)	40.57±2.97 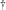	0.967±0.015 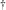	0.922±0.014 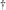	0.904±0.055 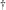
		(1, 2, 4)	40.64±2.90	0.968±0.015	0.923±0.013	0.906±0.052
	24×	(1, 1, 1)	36.01±2.05 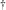	0.941±0.019 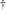	0.886±0.012 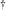	0.804±0.049 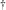
		(1, 3, 5)	36.49±2.24 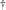	0.945±0.019 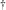	0.892±0.012 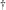	0.822±0.056 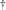
		(1, 2, 4)	36.46±2.14	0.945±0.018	0.892±0.012	0.819±0.052

*Note*: Best results are in **bold**. †: p<0.025 (Bonferroni correction with Wilcoxon signed‐rank test, pairwise comparison against CRUNet‐MR with dilation factor combination of (1, 2, 4)).

Abbreviations: DISTS, deep image structure and texture similarity; HaarPSI, Haar wavelet‐based perceptual similarity index; LAX, long axis; PSNR, peak signal‐to‐noise ratio; SAX; short axis; SSIM, structural similarity.

### 3.7 Evaluation of CRUNet‐MR on the LUMC data

Although CRUNet‐MR achieves promising results on the CMRxRecon2023 dataset, further evaluation on an in‐house dataset remains essential. Unlike the CMRxRecon2023 dataset, the sampling mask of the LUMC in‐house data lacks an ACS region in the center and instead employs strictly uniform sampling lines, which poses a challenge for inference using the current pre‐trained model. Therefore, to thoroughly assess the generalization capability of the pre‐trained CRUNet‐MR model, we designed a three‐step evaluation process: (1) the pre‐trained model was first applied directly to the LUMC data; (2) a new sampling mask consistent with the CMRxRecon2023 dataset, including a 24‐line ACS region, was then generated, and the pre‐trained model was reapplied to evaluate its generalization to data acquired from a different vendor; and (3) to further adapt CRUNet‐MR to the LUMC dataset, we fine‐tuned the model with a limited number of training epochs, allowing it to rapidly achieve satisfactory performance on the in‐house data.

**FIGURE 7:**
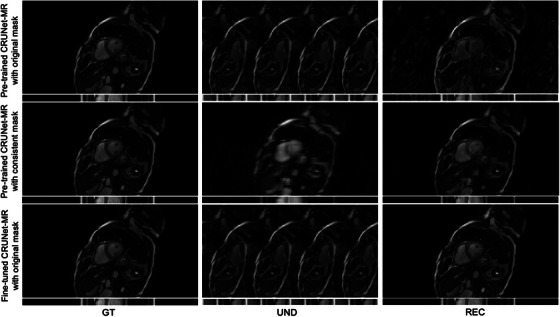
Qualitative evaluation of the generalization ability of CRUNet‐MR on the LUMC data. The first row shows results using the pre‐trained model on the original LUMC data. The second row presents results with a consistent sampling mask, while the last row illustrates reconstructions obtained from the fine‐tuned CRUNet‐MR. Here, ”GT” denotes the ground truth, ”UND” refers to the undersampled visualization obtained using the corresponding mask, and ”REC” represents the reconstructed result.

Then, Table 6 presents the quantitative results of the above three steps on the test set from the fine‐tuning part, while Figure 7 shows qualitative results for a representative test example. These results clearly show that CRUNet‐MR struggles to reconstruct images when confronted with a mismatched sampling mask, which caused the undersampled input images to appear noticeably different. In contrast, with a consistent sampling mask, the pre‐trained model achieves much better reconstructions and demonstrates good generalization to data from a different vendor, although some minor artifacts persist. Moreover, further fine‐tuning on the LUMC dataset enables CRUNet‐MR to adapt rapidly and deliver robust reconstruction performance.

**TABLE 6:** Quantitative evaluation of CRUNet‐MR on the LUMC data.

Steps	PSNR ↑	SSIM ↑
Pre‐trained CRUNet‐MR with original mask	22.50±3.06 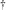	0.537±0.066 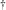
Pre‐trained CRUNet‐MR with consistent mask	31.54±3.25 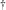	0.849±0.054 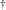
Fine‐tuned CRUNet‐MR with original mask	34.10±3.23	0.953±0.027

Note: Best results are in **bold**. †: p<0.025 (Bonferroni correction with Wilcoxon signed‐rank test, pairwise comparison against fine‐tuned CRUNet‐MR).

Abbreviations: PSNR, peak signal‐to‐noise ratio; SSIM, structural similarity.

## 4 DISCUSSION AND CONCLUSION

In summary, we integrated a deep learning approach into the traditional physics‐based reconstruction formulation for cardiac cine MRI to enhance reconstruction performance through the strong spatio‐temporal representation capabilities of deep learning. By incorporating the proposed deep learning model as a learned regularizer, the method effectively exploits its expressive capacity to capture spatio‐temporal dynamics, enabling more accurate reconstruction at higher acceleration factors, particularly in challenging dynamic cardiac regions.

In the overall comparison among models from Table 1, our proposed CRUNet‐MR outperforms other models across multiple views and acceleration factors, with statistically significant differences in all evaluation metrics. CineNet performed the worst, likely due to its simple U‐Net design and the use of Fourier representations of spatio‐temporal domains (*xt* and *yt*) as inputs of U‐Nets. This design limits its ability to capture spatial features within frames, hindering the learning of spatio‐temporal correlations essential for accurate reconstruction. Despite its simple streamline structure, CRNN‐MRI still outperforms 3D UNet‐MR, highlighting the effectiveness of convolutional recurrent operations in extracting spatio‐temporal features compared to traditional 3D convolutional operations. Similarly, the enhanced performance of CRUNet‐MR over 3D UNet‐MR further underscores this advantage. By comparing CRUNet‐MR with both CRNN‐MRI and 3D UNet‐MR, the benefits of integrating convolutional recurrent operations with a U‐Net structure are further validated. Although PromptMR shows lower overall performance compared to CRUNet‐MR, the performance gap narrows as the acceleration factor increases. As previously mentioned, a core aspect of PromptMR's working principle lies in merging the temporal and channel dimensions and applying a channel attention mechanism to this combined dimension. This design enables the model to effectively extract global spatio‐temporal features, which becomes increasingly important as acceleration factors increase, as more local details are lost due to the removal of high‐frequency components in the *k*‐space domain. However, it is undeniable that PromptMR discards a substantial amount of information from remote frames, which can provide valuable features for the reconstruction. Unlike PromptMR, our proposed CRUNet‐MR processes the entire sequence and uses recurrent operations to link all frames, propagating information across them. The U‐Net structure ensures effective fusion of spatial features, facilitating the exploitation of spatial information. Furthermore, the use of Conv(2+1)D layers, increased dilation factors, and temporal padding collectively enhance the model's ability to extract spatio‐temporal features from the cine sequence. These capabilities enable CRUNet‐MR to achieve superior performance across various acceleration factors and views without relying on a large number of cascade blocks, particularly at lower acceleration factors (e.g., 4 and 8), where finer details are better preserved.

A dynamic analysis was employed to evaluate the models' utilization of spatio‐temporal features by measuring their performance in the challenging dynamic regions, providing a clearer perspective. From Figure 5, CRUNet‐MR demonstrates best performance in reconstructing dynamic regions, highlighting its effective use of spatio‐temporal features. By achieving the highest PSNR values across both views and regions, CRUNet‐MR confirms its superior pixel‐level accuracy. In contrast, PromptMR relies on a neural network to estimate the coil sensitivity map, which might introduce potential inaccuracies that negatively impact pixel values, thereby lowering its PSNR performance. Then, CRUNet‐MR demonstrates highest SSIM values in the dynamic region, while lower SSIM values in the static region compared to PromptMR, except at an acceleration factor of 4. For the reconstruction of dynamic cardiac region, the heart's motion induces substantial pixel intensity variations, disrupting the spatial correlation between pixels, yet it follows a temporal predictable pattern, allowing the model to infer the structural information of the dynamic area by learning this pattern through temporal features. In contrast, the relatively constant pixel intensity values over time rely on stable spatial relationships between neighboring pixels for accurate reconstruction of static region. Additionally, the redundancy between frames in this region allows the extraction of temporal features to further enhance performance. With a limited number of frames, the recurrent operation of CRUNet‐MR effectively extracts global temporal features from the entire sequence, allowing it to capture the overall motion pattern of the heart and enhance the reconstruction of dynamic region. Although CRUNet‐MR's reliance on convolution operations results in a relatively smaller receptive field than PromptMR, potentially limiting its ability to capture global spatial information, the use of dilation factors helps expand the receptive field, mitigating this limitation by enhancing spatial feature extraction. Overall, CRUNet‐MR still demonstrates greater effectiveness in leveraging spatio‐temporal features compared to the other methods.

Evaluation of CRUNet‐MR on the LUMC in‐house data highlights the limited generalization capability of deep learning‐based reconstruction methods across different sampling masks. When the sampling mask is altered, thereby changing the distribution of the model input, CRUNet‐MR struggled to achieve satisfactory reconstruction. In contrast, when the input distribution is preserved, the model can generalize to data acquired from different vendors, albeit with a slight performance degradation. Fine‐tuning CRUNet‐MR on a small LUMC dataset, however, enabled strong performance on the in‐house data. Based on the CMRxRecon2024 challenge paper,^28^ a straightforward solution to achieve robust reconstruction across varying sampling masks, is to incorporate training data from different sampling masks, allowing deep learning methods such as CRUNet‐MR to reconstruct effectively in multiple scenarios.

While CRUNet‐MR has made big progress in utilizing spatio‐temporal features, there still remains room for improvement and exploration. First, in the CMRxRecon2023 and the LUMC dataset, a fixed sampling mask is applied across all frames. Adopting dynamic sampling masks could capture more diverse *k*‐space information and make CRUNet‐MR better exploit spatio‐temporal features, potentially achieving better reconstruction performance. Secondly, effectively capturing global spatio‐temporal features is crucial, as highlighted by the above results and analysis. Although PromptMR performs well by applying attention across five frames, extending this approach to an entire sequence is highly more challenging. The simplicity of channel attention limits its ability to capture complex patterns with more frames, while potential artifact interference from remote frames may further degrade performance. Additionally, this extension would substantially increase GPU memory requirements. In contrast, convolutional recurrent operations establish strong frame‐to‐frame connections and effectively leverage spatio‐temporal features for reconstructing dynamic regions, enabling the simultaneous processing of more frames and making them well‐suited for cine MRI reconstruction. However, their intrinsic limitations, particularly when handling longer sequences, can restrict their capacity to effectively model global spatio‐temporal dependencies. Moreover, convolutional recurrent operations also demand substantial GPU memory, as each frame requires a separate convolution operation, thereby constraining the channel number and network depth of the U‐Net structure compared to PromptMR. Consequently, enhancing the efficiency of spatio‐temporal feature extraction to enable more complex model architectures, along with improving the CRUNet‐MR framework's ability to capture global spatio‐temporal features, represents a promising direction for future research.

In this work, we proposed CRUNet‐MR, a model optimized for spatio‐temporal feature extraction in 2D cardiac cine reconstruction. By integrating a split bidirectional convolutional recurrent unit into a U‐Net structure, CRUNet‐MR effectively leverages spatio‐temporal features across the entire sequence. Benchmark comparisons and dynamic analysis validate its enhanced spatio‐temporal information utilization. Additionally, the investigation of dilation factors highlights the importance of expanding receptive field, especially for high acceleration factors. Although CRUNet‐MR exhibits limited generalization across different sampling masks, it maintains robust performance across data from different vendors and can rapidly adapt through fine‐tuning. Overall, we believe that the design of CRUNet‐MR is well‐suited for cine MRI reconstruction, offering the potential to push acceleration limits and support practical clinical applications.

## CONFLICT OF INTEREST STATEMENT

The authors have no relevant conflicts of interest to disclose.
